# Nitrogen fertilizer application rate affects the dynamic metabolism of nitrogen and carbohydrates in kernels of waxy maize

**DOI:** 10.3389/fpls.2024.1416397

**Published:** 2024-08-01

**Authors:** Wanjun Feng, Weiwei Xue, Zequn Zhao, Zhaokang Shi, Weijie Wang, Yu Bai, Haoxue Wang, Peng Qiu, Jianfu Xue, Baoguo Chen

**Affiliations:** ^1^ Sorghum Research Institute, Shanxi Agricultural University, Yuci, Shanxi, China; ^2^ College of Agriculture, Shanxi Agricultural University, Taigu, Shanxi, China

**Keywords:** waxy corn, nitrogen application, protein components, carbohydrate content, enzyme activity

## Abstract

**Introduction:**

Nitrogen (N) plays a pivotal role in the growth, development, and yield of maize. An optimal N application rate is crucial for enhancing N and carbohydrate (C) accumulation in waxy maize grains, which in turn synergistically improves grain weight.

**Methods:**

A 2-year field experiment was conducted to evaluate the impact of different N application rates on two waxy maize varieties, Jinnuo20 (JN20) and Jindannuo41 (JDN41), during various grain filling stages. The applied N rates were 0 (N0), 120 (N1), 240 (N2), and 360 (N3) kg N ha^-1^.

**Results:**

The study revealed that N application significantly influenced nitrogen accumulation, protein components (gliadin, albumin, globulin, and glutelin), carbohydrate contents (soluble sugars, amylose, and amylopectin), and activities of enzymes related to N and C metabolism in waxy maize grains. Notable varietal differences in these parameters were observed. In both varieties, the N2 treatment consistently resulted in the highest values for almost all measured traits compared to the other N treatments. Specifically, the N2 treatment yielded an average increase in grain dry matter of 21.78% for JN20 and 17.11% for JDN41 compared to N0. The application of N positively influenced the activities of enzymes involved in C and N metabolism, enhancing the biosynthesis of grain protein, amylose, and amylopectin while decreasing the accumulation of soluble sugars. This modulation of the C/N ratio in the grains directly contributed to an increase in grain dry weight.

**Conclusion:**

Collectively, our findings underscore the critical role of N in regulating kernel N and C metabolism, thereby influencing dry matter accumulation in waxy maize grains during the grain filling stage.

## 1 Introduction

Waxy maize (*Zea mays* L. var. *ceratina* Kulesh) is a subspecies of maize, also called sticky maize, and is characterized by its viscosity and digestibility. It emerged in southwest China in the early 20th century (Collins, 1909). Due to the mutation of the *waxy* gene (*Wx*), almost all the starch synthesized in the kernels of waxy corn is amylopectin (Schwartz and Whistler, 2009). In recent decades, waxy maize has gained popularity among consumers and farmers in Asia due to its unique taste, high nutritional and economic value, and ease of processing (Yang et al., 2021). In addition, it is also an important industrial raw for the textile, adhesive, brewing and paper industries (Klimek-Kopyra et al., 2012; Liu et al., 2021).

Nitrogen (N) is an essential mineral element for plants and an important regulatory factor for plant growth, development and yield production (Kant, 2018). In fact, the increasing use of N fertilizers over the last half century has contributed significantly to significantly improving global food production (Foley et al., 2011). However, excessive N application often occurs in crop production around the world, resulting in lower N use efficiency (NUE) for plants (Zhang et al., 2015). According to field research experiments, the NUE ranges from 10 and 60%, which is lower than other cereals (Cui et al., 2010; Ladha et al., 2016; Morris et al., 2018). In addition, large N fertilizer loss also leads to adverse effects on crop quality, soil acidification, greenhouse effect, environmental pollution and human health (Guo et al., 2010; Mueller et al., 2012; Liu et al., 2020). Therefore, to address the triple challenges of climate change, environmental degradation and food security, increasing crop NUE can be a useful tactic (Zhang et al., 2015).

Nitrogen and carbohydrate metabolism in corn kernels is closely related to the synthesis and accumulation of storage proteins, starch, and other compounds. Compared to other maize, waxy maize lacks the production of amylose, resulting in different carbohydrate metabolism as starch composed vast majority of amylopectin. Meanwhile, previous studies demonstrated that N and carbohydrate metabolism have interaction in maize kernels (Swank et al., 1982; He et al., 2004; Li et al., 2022). Hence, it is believed that carbon and nitrogen metabolism in kernels between waxy maize and other maize may be significant differently. Starch and protein are two important nutritional components of cereal grains, and the dynamics of their content and accumulation influence grain yield and quality (Lu et al., 2014; Zhang et al., 2021). The process of starch and protein biosynthesis in cereal grains is complicated and relies on the synchronized activity of lots of enzymes (Ran et al., 2020). In the process of N assimilation, nitrate reductase (NR), nitrite reductase (NiR), glutamine synthetase (GS), and glutamine-2-oxoglutarate aminotransferase (GOGAT, also known as glutamate synthase) are the most important enzymes (Liu et al., 2022). Soluble starch synthase (SSS), granule bound Starch Synthase (GBSS), starch branching enzyme (SBE) and starch debranching enzyme (SDBE) are involved in starch synthesis in cereal grains and play crucial roles in the metabolic processes of grain development and yield formation, and ADP-glucose pyrophosphorylase (AGPase) is responsible for converting ADP-glucose into starch polymers, providing energy and building blocks for grain development (Smith and Zeeman, 2020).

It is clear that N fertilization affects the nitrogen metabolism in plant, influencing the synthesis of proteins and amino acids, as well as the translocation and allocation of N in maize (Chen et al., 2015; Mueller and Vyn, 2016). It has long been suggested that grain protein content (GPC) can be increased with an appropriate amount of N fertilizer (Qi et al., 2006). Furthermore, N availability can also impact the photosynthetic activity and carbohydrate metabolism in grains, thereby influencing the accumulation of starch and other carbohydrates is crucial for kernel development and yield formation (Mueller and Vyn, 2016). However, the specific impacts on the dynamic metabolism of nitrogen and carbohydrates in waxy maize kernels remain relatively understudied. Understanding the intricate biochemical and physiological processes underlying the response of waxy maize kernels to varying N fertilizer application rates is crucial for optimizing agricultural practices and enhancing waxy maize yield and quality. In this study, we aim to investigate the effects of nitrogen fertilization on the metabolism of nitrogen and carbohydrates in waxy maize kernels at different days after pollination (DAP), with a focus on elucidating the regulatory mechanisms that govern waxy maize yield, to provide valuable insights into the physiological and biochemical basis for optimizing nitrogen management strategies to maximize waxy maize yield and quality.

## 2 Materials and methods

### 2.1 Material planting and preparing

Two waxy maize varieties (Jinnuo20 (JN20), a purple waxy maize variety; and Jindannuo41 (JDN41), a yellow waxy maize variety) were used as experimental materials, the seeds of which were purchased from Shanxi Dafeng Seed Industry Co., LTD. The field experiment was conducted in Dongshan bottom Village (37°22'28" N, 12°35'8" E), Taigu County, Shanxi Province, China, in 2018 and 2019. The field had been planted with trees for five years without planting crops or fertilizing before the trial began. Prior to conducting the experiment, the tested topsoil (0–20 cm) was sandy loam with pH value of 6.1, and the soil nutrient content prior to sowing was as follows: the contents of total N, alkaline hydrolyzable N, available phosphorus (P), exchangeable potassium (K) and organic substances were 0.66 g kg^-1^, 30.24 mg kg^-1^, 20.02 mg kg^-1^, 114.11 mg kg^-1^ and 18.10 g kg^-1^, respectively.

The experiment employed a split-plot design, incorporating three replicates. Main plots were allocated to two varieties of waxy maize, while subplots were designated to four nitrogen (N) fertilizer application rates: 0, 120, 240, and 360 kg ha^-1^ of pure N, referred to as N0, N1, N2, and N3, respectively. Maize planting occurred in late May for two consecutive years, utilizing a randomized plot arrangement with triple replicates. Each plot measured 40 m^2^ (4 m x 10 m) and contained 8 rows, with a planting density of 60,000 plants ha^-1^. Fertilizers used included urea (46% N) for nitrogen, superphosphate (12% P_2_O_5_) for phosphorus, and potassium chloride (60% K_2_O) for potassium. Nitrogen was applied in a 3:5:2 ratio at the jointing, booting, and anthesis-silking stages. Additionally, 120 kg ha^-1^ of pure phosphorus (P_2_O_5_) and 240 kg ha^-1^ of pure potassium (K_2_O) were administered in a 1:1 ratio at sowing and jointing stages. Corn cultivation followed standard field management practices.

The harvested ears were manually bagged to ensure pollination occurred without external pollen contamination. Sampling was conducted from 15 to 35 days after pollination (DAP), at five-day intervals. In each of the three replicates across all treatment groups, five uniformly growing ears were selected for seed extraction. Some seeds were blanched in an oven at 120 ° for 30 min, and dried at 80 ° to constant weight, and used to determine the biomass, nitrogen content, protein content and its fractions, and soluble sugar and starch content of the other seeds frozen in liquid nitrogen and quickly stored in an ultra-low temperature refrigerator of -80°C for measuring the activity of N and carbohydrate metabolic enzymes. All measurements were taken in three biological replicates.

### 2.2 Total nitrogen content assay

Total nitrogen content in the seeds was measured by a modified Kjeldahl digestion method (Nelson and Sommers, 1962). In brief, 0.5 g of dry grain flour was deboiled by H_2_SO_4_ for 10 min, and then heated for 5-10 min to remove the remaining H_2_O_2_. Later, the decocting liquid was fixed to 100 mL with distilled water, and 1.0 mL of the diluted solution was absorbed and added 1 mL EDTA-methyl red solution adjusting with NaOH (0.3 mo1 L^-1^) to pH value for 6.0, and 5 mL phenol solution and 5 mL sodium hypochlorite solution were added in turn. After 1 h, the optical density (OD) was measured using a UV spectrophotometer (Shanghai Metash Instruments Co., Ltd., Shanghai, China) at 625 nm. The blank test solution was used to adjust zero point of instrument absorption value. After measuring the absorption value, the working curve was drawn to calculate the nitrogen content of samples.

### 2.3 Protein quantification

Protein content was assayed using the Coomassie brilliant blue G-250 method (Li and Li, 2000). Initially, a Coomassie brilliant blue G-250 and standard protein solutions were prepared. Using a mortar and pestle, 0.5 g of dry seeds were ground with 10 mL of distilled water. After centrifugation of the solution at 4000 × g at 4°C for 10 min, the supernatant was transferred to a clean tube and total protein content measured as the change in absorbance at 595 nm. Then, prepare a BSA protein standard at 1 mg mL^-1^ concentration in duplicate and dilute the protein standard in a volume of 20 μL to give 5 concentrations over a range of 10 to 50 μg protein. Add 20 μL of protein solution to 1 mL of dye reagent, mix, incubate for 2 min at room temperature and measure the absorbance in a cuvette. Subsequently, a calibration curve was plotted to depict the relationship between the absorbance values at 595 nm and the known protein concentrations. The protein concentration of the unidentified sample was deduced by aligning its absorbance value with the established calibration curve.

### 2.4 Determination of protein components

Protein components were determined using a previous method with slight modifications (Ju et al., 2001). 0.5 g fresh sample was ground into a homogeneous slurry in an ice bath and transferred to a 5 mL centrifuge tube. The slurry was then mixed with 5 mL of distilled water and agitated for 30 min. The mixture was centrifuged at 2000 rpm at 4 ˚C for 5 min, after which the supernatant was decanted, and the pellet was resuspended in 5 mL of distilled water for precipitation. This process was repeated twice, and the collected supernatant was adjusted to a final volume of 50 mL for the purpose of albumin quantification. Subsequently, 5 mL of a 10% sodium chloride solution was added to the residue within the centrifuge tube to facilitate albumin extraction. After the subsequent extraction method of albumin, centrifuge at 3000 g for 0.5 h and repeat three times to obtain the globulin fraction in the supernatant. The pellet from the previous step was dissolved with 5 mL of NaCl solution (10%) and centrifuge at 3000 g for 0.5 h at 4 ˚C. Following this centrifugation, the albumin extraction methodology was employed, and the procedure was repeated twice to further isolate the globulin component from the supernatant. For the extraction of the glutelin fraction, the precipitate remaining in the centrifuge tube was treated with 0.1 M NaOH (400 mL) at room temperature and subsequently centrifuged at 3000 g for 30 min. To isolate the gliadin fraction, the precipitate was then extracted with 70% ethanol (400 mL) under ambient conditions and centrifuged at 3000 g for 30 min.

The quantification of the extracted total proteins, albumins, globulins, gliadins, and glutelins was performed utilizing a UV spectrophotometer, with Bovine Serum Albumin (BSA) serving as the calibration standard. To establish a calibration curve, six standard solutions were prepared, comprising 0, 2.5, 5, 10, 15, and 20 μL of a BSA stock solution (1 mg mL^-1^) each diluted in 1 mL of Bradford reagent. For the assay, 2 μL of each protein extract was combined with 1 mL of Bradford reagent. The tubes were then thoroughly mixed by inversion before the absorbance was measured at 595 nm using a UV spectrophotometer (Shanghai Metash Instruments Co., Ltd., Shanghai, China) (Marion, 1976). The standard linear curve of six points was created by using MS excel and concentrations of protein samples were calculated.

### 2.5 Starch content

The amylose content and amylopectin content were spectrophotometrically determined by the double-wavelength method (Dong et al., 2003). For the determination, 0.1 g of milled grains were stirred with 10 mL of 0.5 M KOH for 30 min at 90°C and then diluted to a volume of 50 mL with distilled water. From this, 2.5 ml was removed to a fresh tube containing 20 mL distilled water. The solution was adjusted to pH 3.5 with 0.1 M HCl, and 500 μL of I_2_-KI reagent added. Finally, this solution was diluted to a final volume of 50 mL with distilled water. After standing for 20 min, the absorbance of the mixture was measured with a UV spectrophotometer (Shanghai Metash Instruments Co., Ltd., Shanghai, China) at 480, 550, 630, and 735 nm, respectively. The total starch content was defined as the sum of amylose and amylopectin content.

### 2.6 Soluble sugars content

Soluble sugars were extracted according to a previous method (Adney and Baker, 1996). After being ground and homogenized with 10 mL of deionized water, 500 mg samples along with 5 mL of an 80% ethanol (C_2_H_6_O) solution were placed in a water bath at 45°C for 20 minutes. Following this, the samples were allowed to cool to room temperature. The homogenate was then centrifuged twice at 6000 rpm for 10 minutes at 15°C. Next, 2 mL of the supernatant was combined with 2 mL of 3,5-dinitrosalicylic acid reagent, thoroughly mixed, and then boiled in a water bath for 5 min. After boiling, the mixture was cooled to room temperature in a water-ice bath. The supernatant was collected for the determination of soluble sugar content using a UV spectrophotometer at 540 nm (Shanghai Metash Instruments Co., Ltd., Shanghai, China). Distilled water was used as a control, and glucose was employed to create a standard curve. The C/N ration was calculated by the formula: C/N ration  = (Soluble sugar content + Starch content)/Total nitrogen content.

### 2.7 Enzyme activities

Nitrate reductase (NR) was extracted and measured using a Nitrate Reductase (NR) Assay Kit (BC0080, Solarbio, Beijing, China). In brief, 0.1 g of fresh seed powder that frozen and grinded in liquid nitrogen was extracted in 1 mL extraction solution, and the mixture was centrifuged at 4000 × g at 4 ° for 10 min. The supernatant was absorbed for analyzing the OD value, and the absorbance at 520 nm was used for the calculation of NR activity.

Glutamine synthetase (GS) was extracted and measured using a Glutamine Synthetase (GS) Assay Kit (BC0915, Solarbio, Beijing, China). Briefly, 0.1 g of fresh seed powder frozen and ground in liquid nitrogen was extracted with 1 mL extraction buffer. The mixture was centrifuged for 10 min at 8000 × g at 4°C. Finally, the supernatant was collected after centrifugation for analysis of OD value and the absorbance at 520 nm was used to calculate GS activity.

The preparation procedure was according to a previous method (Nakamura et al., 1989). For the assays of enzymes, 5-10 g frozen grains were weighed and homogenized with a pestle in a pre-cooled mortar containing 10 mL ice-cooled extraction buffer (50 mM Hepes-NaOH [pH 7.5], 2 mM KCl, 5 mM EDTA, 1 mM DTT [Dithiothreitol], 1% (w/v) PVP [polyvinylpyrrolidone-30]). An aliquot of the homogenate (30 μL) was mixed with 1.8 mL extraction buffer and then centrifuged at 2000 g at 4°C. The homogenate was centrifuged at 10000 g for 0.5 h at 4°C, and the resulting supernatant was used for the determination of AGPase, SSS, SBE and DBE activities. The precipitate was re-suspended in 0.5 mL extraction solution and used for the determination of GBSS activity. Meanwhile, 50.0 μL of crude enzyme solution was taken and added to boiling water for 60 s in advance. The check procedure was then carried out as above. The production of NADH was monitored spectrophotometrically at 340, 540 and 660 nm using a UV spectrophotometer (Shanghai Metash Instruments Co., Ltd., Shanghai, China).

### 2.8 Data analysis

Statistical analysis was performed using Excel (Microsoft Office 2016) and SPSS v.26 statistical package (SPSS, Chicago, IL, USA). The test of normal distribution was conducted using the “nortest” packages in Rstudio software. Differences of above testing parameters, as affected by N application rate, harvest period, year and their interactions, were examined using a three-factor model of analysis of variances (ANOVA). When the ANOVA was proved significant for any parameter, a least significant difference (LSD) test was assayed for multiple comparisons at *P ≤* 0.05. Correlation analysis was performed using the Origin2022 software (OriginLab Corporation, Northampton, MA, USA). Regression analysis was performed using the “ggplot2” and “ggsignif” packages in Rstudio software. A structural equation model (SEM) was used to analyze the effects of N fertilizer application on detected characteristics of waxy maize grains using the “lavaan” package in Rstudio software. Data in figures were the average of three biological replicates.

## 3 Results

### 3.1 Effects of nitrogen level on the dynamic accumulation of kernel dry matter

**Figure 1:**
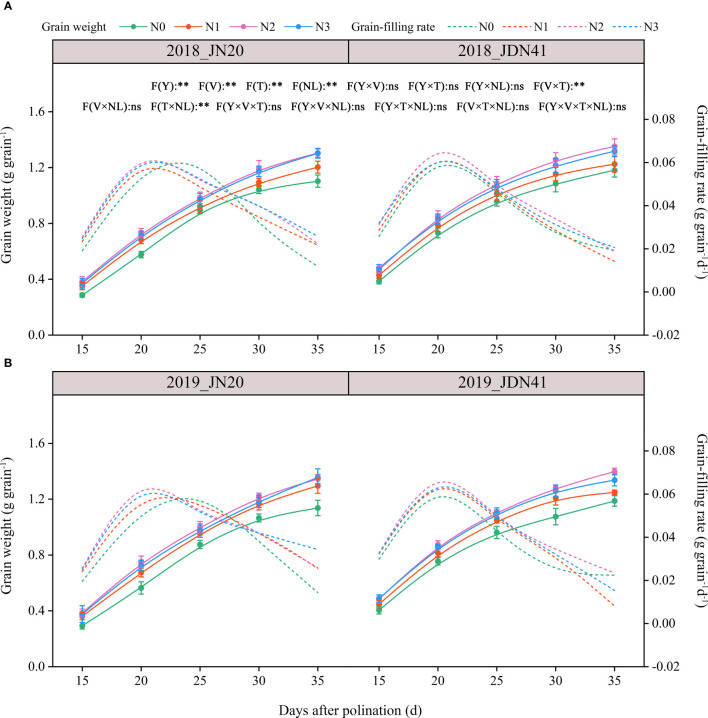
Effects of nitrogen rate on the grain weight dynamics and filling rate of two waxy maize varieties at different days after pollination in 2018 **(A)**, 2019 **(B)**. The grain filling rate is the derivative of the grain weight accumulation. ns indicates no significant differences, and * and ** indicate significant differences at *P* < 0.05, and *P* < 0.01 level, respectively. The error bar indicates standard error based on 3 data.

The ANOVA analysis indicated that grain dry weight (GDW) was significantly affected by year (Y), filling time (T), variety (V), nitrogen level (NL), V×T and T×NL, but other interactions between the two or three factors had no significant effects on the GDW (**Figure 1**; **Supplementary Figure 1**). The effects of four nitrogen levels on GDW of Jinnuo20 (JN20) and Jindannuo 41 (JDN41) at different days after pollination (DAP) were similar and consistent in two years, all showing N2≥N3>N1>N0. In detail, the JN20 GDW under N1, N2 and N3 treatments raised with ranges of 1.11%-40.04%, 5.95%-55.80% and 3.72%-58.07% compared to N0 at different DAP over the two years, respectively. And the GDW of JDN41 under N1, N2 and N3 treatments increased with ranges of 2.67%-24.43%, 4.69%-33.00% and 3.05%-33.45% compared to N0 based on the five filling time points within the two years, respectively. Furthermore, the GDW of JN20 and JDN41 both raised gradually from 15 to 35 DAP under four N treatments, and the GDW of JN20 and JDN41 increased by an average of 94.68% and 81.83%, 173.26% and 133.43%, 229.60% and 166.67%, and 261.55% and 186.38% at 20, 25, 30, and 35 DAP, compared to 15 DAP, respectively, over the four N treatments within two years.

Obviously, nitrogen level also had significant effect on grain filling rate (GFR) of JN20 and JDN41 (**Figure 1**; **Supplementary Figure 1**). The GFR of JN20 under N1, N2 and N3, and that of JDN41 under N0, N1, N2 and N3, all reached a peak at 20 DAP, but that of JN20 under N0 delayed to 25 DAP, indicating that nitrogen levels had different effects on the GFR within maize varieties. Except for JN20 at 25 DAP, which was higher under N0 than other three N treatments, the GFR of JD20 under N1, N2 and N3 treatments all enhanced with ranges of 2.22% (15 DAP)-1129.13% (35 DAP), 2.20% (15 DAP)-891.26% (35 DAP) and 1.37% (15 DAP)-659.22% (35 DAP) compared to N0 over the five filling time points within the two years, respectively. For JDN41, the GFR at 35 DAP showed the higher values under N0 compared with other N treatments, but that of JDN41 at other grain filling stages all increased with ranges of 5.38%-104.08%, 7.30%-167.53% and 5.14%-119.00%, respectively.

### 3.2 Effects of nitrogen level on the dynamic metabolism of grain nitrogen

#### 3.2.1 Grain nitrogen content

**Figure 2:**
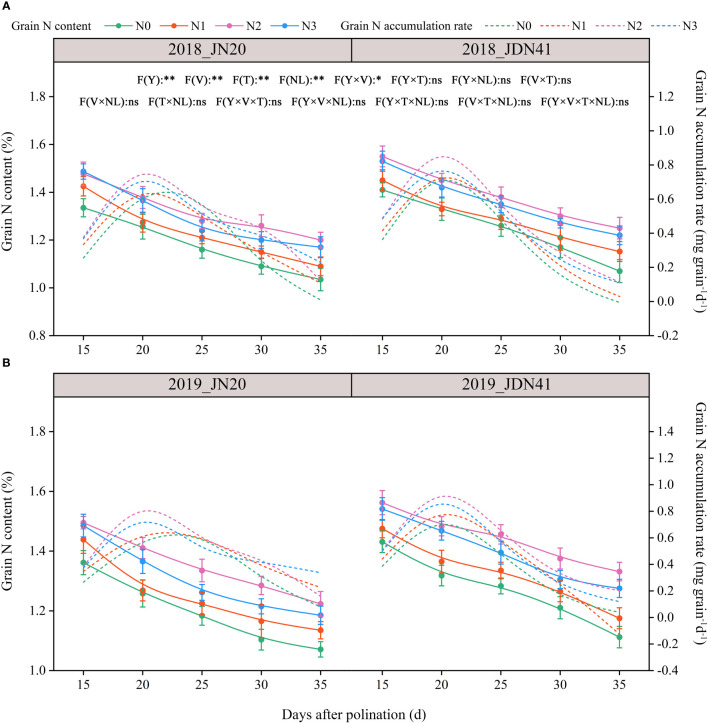
Effects of nitrogen rate on the grain N content and accumulation rate of two waxy maize varieties at different days after pollination in 2018 **(A)**, 2019 **(B)**. The grain N accumulation rate is the derivative of the grain N content accumulation. ns indicates no significant differences, and * and ** indicate significant differences at *P* < 0.05, and *P* < 0.01 level, respectively. The error bar indicates standard error based on 3 data.

The GNC was significantly affected by factors Y, T, V, NL, and Y×V, but the other interactions between the two or three factors had no significant effects on the GNC (**Figure 2**; **Supplementary Figure 2**). The effects of four nitrogen levels on GNC of JN20 and JDN41 at different DAP were similar and constant within the two years, almost showing a trend of N2>N3>N1>N0. The JN20 GNC under N1, N2, and N3 treatments grew by different ranges compared to N0 over two years. Specifically, the increases were between 0.26% and 14.46%, 4.22% and 21.58%, and 1.82% and 14.67%. Similarly, the JDN41 GNC increased by ranges of 1.29% to 14.81%, 5.13% to 24.49%, and 4.21% to 18.50%, respectively. In addition, the GNC of JN20 and JDN41 both dropped gradually from 15 to 35 DAP under four N treatments, which decreased by an average of 8.03% and 6.57%, 13.96% and 10.06%, 17.72% and 15.45%, and 20.84% and19.85% at 20, 25, 30, and 35 DAP compared to 15 DAP, respectively, over the four N treatments within two years.

In consistent with GFR, the grain N accumulation rate (GNAR) of JN20 under N1, N2 and N3, and that of JDN41 under four nitrogen levels, all reached a peak close to 20 DAP, but that of JN20 under N0 delayed close to 25 DAP (**Figure 2**; **Supplementary Figure 2**), indicating that nitrogen level also had significant effect on GNAR of both JN20 and JDN41, but with different effects within varieties. For instance, the GNAR of JN20 and JDN41 had the greatest values under N2 treatment, increasing by an average of 431.21% and 131.39% compared to N0 during the five filling time points over the two years.

#### 3.2.2 Nitrate reductase and glutamine synthetase activity

**Figure 3:**
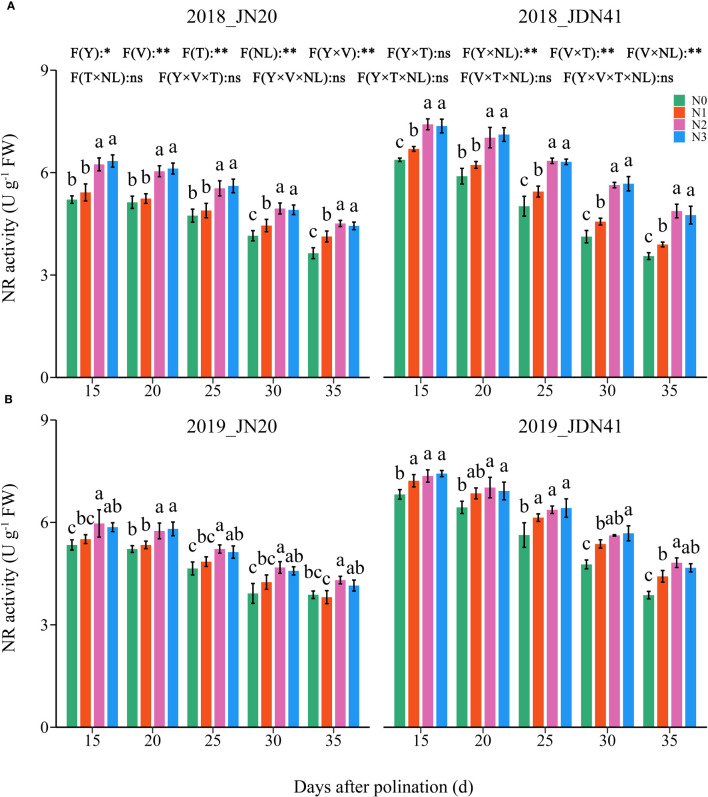
Effects of nitrogen rate on nitrate reductase (NR) activity of two waxy maize varieties at different days after pollination in 2018 **(A)**, 2019 **(B)**. ns indicates no significant differences, and * and ** indicate significant differences at *P* < 0.05, and *P* < 0.01 level, respectively. The different small letters above the error bars indicate significant differences between different treatments at the *P* < 0.05 level. The error bar indicates standard error based on 3 data.

As shown in **Figure 3**, the NR activity in JDN41 grains was much higher than that of JN20 under the same N level and at the same time point. The NR activity in the grains of both genotypes decreased gradually from 15-35 DAP under four N application rates within two years. The effects of N level on NR activity of both genotypes at different DAP were similar and consistent within two years. At any individual time point, the NR activity in the grains of both genotypes showed N3≥N2>N1≥N0. In detail, the NR activity of JN20 rose by 0.25%-23.12%, 4.58%-34.26%, and 4.06%-30.64% after N1, N2, and N3 treatments compared to N0 over the two-year period. And JDN41’s values increased by 1.01%-16.80%, 2.88-45.93%, and 3.59%-45.64%, respectively (**Supplementary Figure 3**).

**Figure 4:**
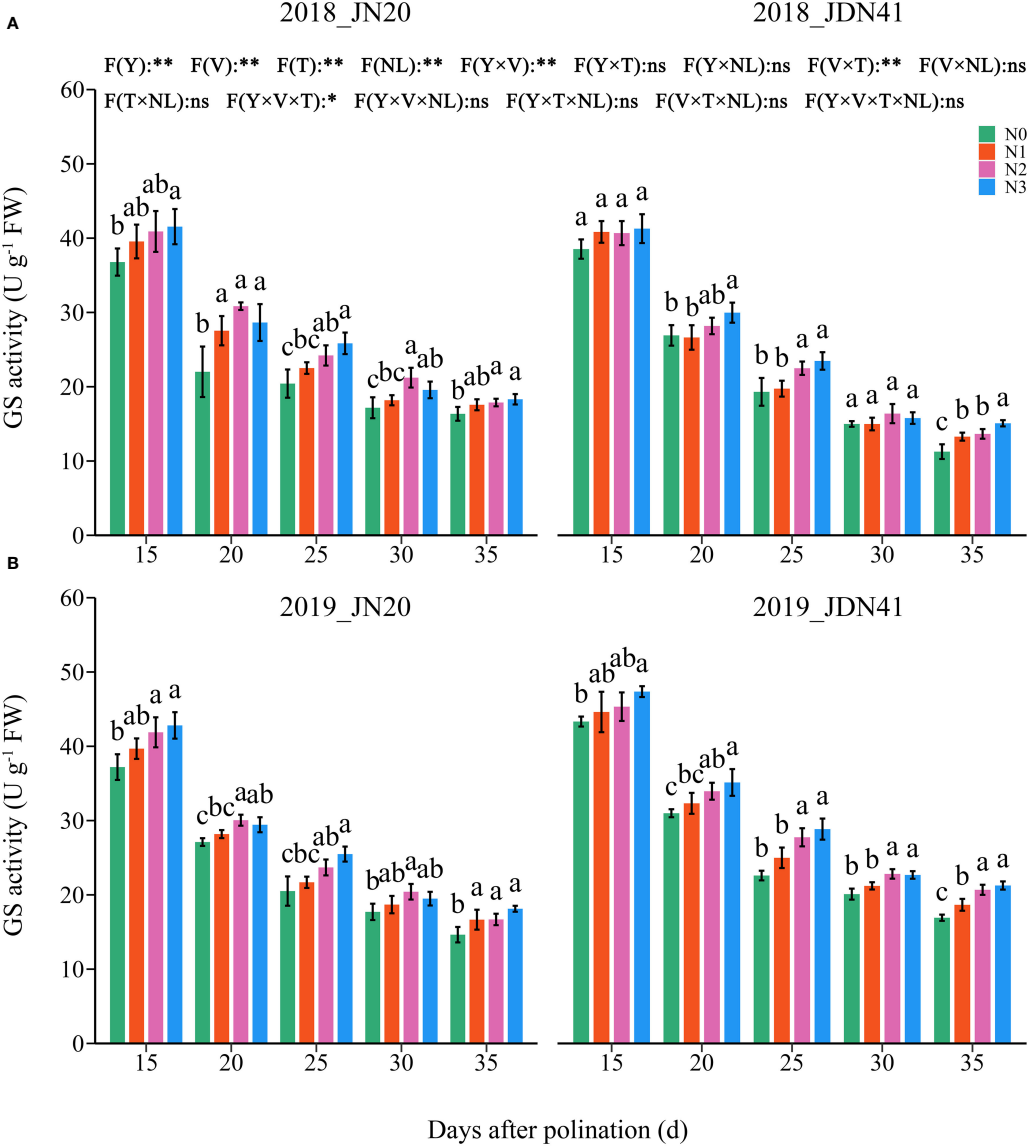
Effects of nitrogen rate on glutamine synthetase (GS) activity of two waxy maize varieties at different days after pollination in 2018 **(A)**, 2019 **(B)**. ns indicates no significant differences, and * and ** indicate significant differences at *P* < 0.05, and *P* < 0.01 level, respectively. The different small letters above the error bars indicate significant differences between different treatments at the *P* < 0.05 level. The error bar indicates standard error based on 3 data.

In contrast, the GS activity was unobvious between the two genotypes under the same N level and at the same time point (**Figure 4**; **Supplementary Figure 3**). Similarly, the GS activity in the grains of both genotypes decreased gradually from 15-35 DAP under four N levels within two years. The effects of N level on GS activity of both genotypes at different DAP were similar and consistent within two years. At the same grain filling stage, the GS activity in the grains of both genotypes showed a trend of N3≥N2>N1≥N0. In detail, the GS activity of JN20 under N1, N2 and N3 treatments increased by an average of 6.04%, 11.44% and 14.94% compared to N0 over five time points within two years, respectively. And that of JDN41 increased by an average of 6.52%, 12.80% and 16.49%, respectively. Above results indicated that increasing N rate would induce the NR and GS activity.

#### 3.2.3 Protein components content

**Figure 5:**
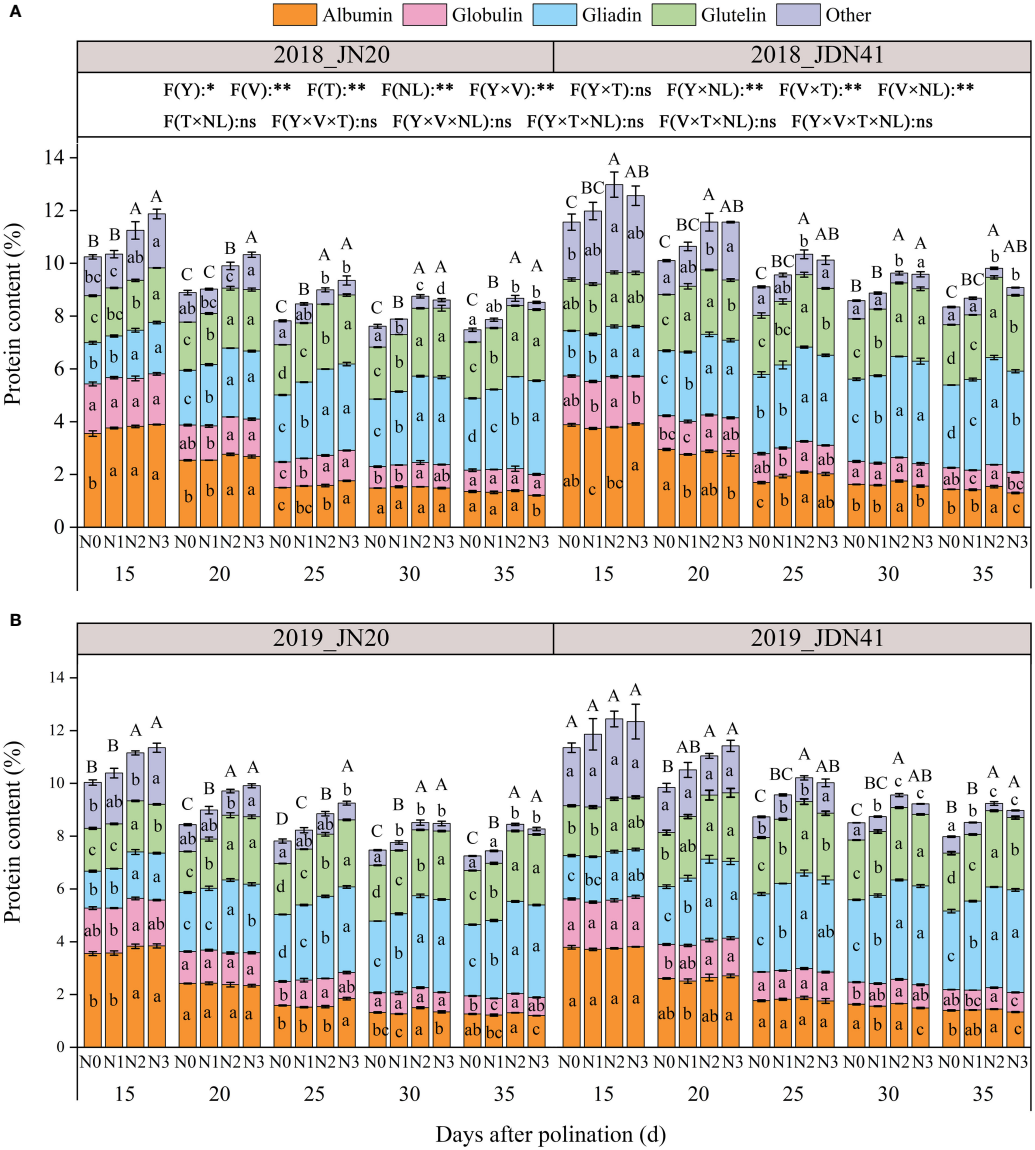
Effects of nitrogen rate on protein content and components of two waxy maize varieties at different days after pollination in 2018 **(A)**, 2019 **(B)**. ns indicates no significant differences, and * and ** indicate significant differences at *P* < 0.05, and *P* < 0.01 level, respectively. Different lowercase and capital letters indicate significant differences in protein components and protein content between different treatments at the *P*<0.05 level, respectively. The error bar indicates standard error based on 3 data.

The protein components in maize kernels are complex, which classified as gliadin (also called zeins), albumin, globulin and glutelin according to their solubility. As shown in **Figure 5**, the total protein content of JDN41 was higher than that of JN20 at any time point under four nitrogen levels in two years. Over time, the levels of albumin, globulin, and other protein components of both genotypes gradually decreased under the four nitrogen levels. On the contrary, both gliadin and glutelin content of the two genotypes showed a gradually increasing trend. The effects of N level on all protein components of both genotypes at any grain filling stage were almost coincident within two years, all showing N2≥N3>N1>N0. In detail, the grain albumin content of JN20 under N0, N1, N2 and N3 decreased from 3.55%, 3.67%, 3.82% and 3.87% at 15 DAP to 1.31%, 1.27%, 1.35% and 1.20% at 35 DAP over the two years, while that of JDN41 declined from 3.84%, 3.73%, 3.77% and 3.86% at 15 DAP to 1.42%, 1.42%, 1.49% and 1.32% at 35 DAP, respectively (**Figure 5**; **Supplementary Figure 4**). For the grain globulin content of JN20, it diminished from 1.80%, 1.80%, 1.82% and 1.83% at 15 DAP to 0.74%, 0.75%, 0.78% and 0.74% at 35 DAP, while that of JDN41 declined from 1.84%, 1.78%, 1.87% and 1.85% at 15 DAP to 0.80%, 0.75%, 0.82% and 0.76% at 35DAP under N0, N1, N2 and N3, respectively. However, the grain gliadin content of JN20 under N0, N1, N2 and N3 improved from 1.48%, 1.54%, 1.79% and 1.86% at 15 DAP to 2.71%, 2.99%, 3.49% and 3.53% at 35 DAP over the two years, while that of JDN41 promoted from 1.68%, 1.75%, 1.88% and 1.84% at 15 DAP to 3.06%, 3.40%, 3.94% and 3.86% at 35 DAP, respectively. The grain glutelin content of JN20 increased from 1.70%, 1.76%, 1.91% and 1.96% at 15 DAP to 2.09%, 2.25%, 2.68% and 2.68% at 35 DAP, while that of JDN41 decreased from 1.92%, 1.89%, 2.02% and 2.01% at 15DAP to 2.24%, 2.49%, 2.96% and 2.81% at 35DAP, respectively. These results demonstrated that the albumin and globulin were preferentially synthesized at the early grain filling stage, while gliadin and glutelin were mainly accumulated at the later grain filling stage, and N had significant effects on the synthesis of protein components, especially on gliadin and glutelin synthesis.

#### 3.2.4 Regression analysis

Both JN20 and JDN41 GNC were significantly positively correlated with the NR activity (R^2^ of JN20 and JDN41 was 0.74 and 0.84), GS activity (R^2^ of JN20 and JDN41 was 0.78 and 0.68) (**Supplementary Figure 5**), albumin content (R^2^ of JN20 and JDN41 was 0.72 and 0.61) and globulin content (R^2^ of JN20 and JDN41 was 0.72 and 0.66) (**Supplementary Figure 6**). On the contrary, both JN20 and JDN41 GNC were significantly negatively correlated with the gliadin content (R^2^ of JN20 and JDN41 was 0.30 and 0.28), and the relationship between GNC and glutelin content of both genotypes was nonlinear (**Supplementary Figure 6**).

### 3.3 Effects of nitrogen level on the dynamic metabolism of grain carbohydrates

#### 3.3.1 Carbohydrate content

**Figure 6:**
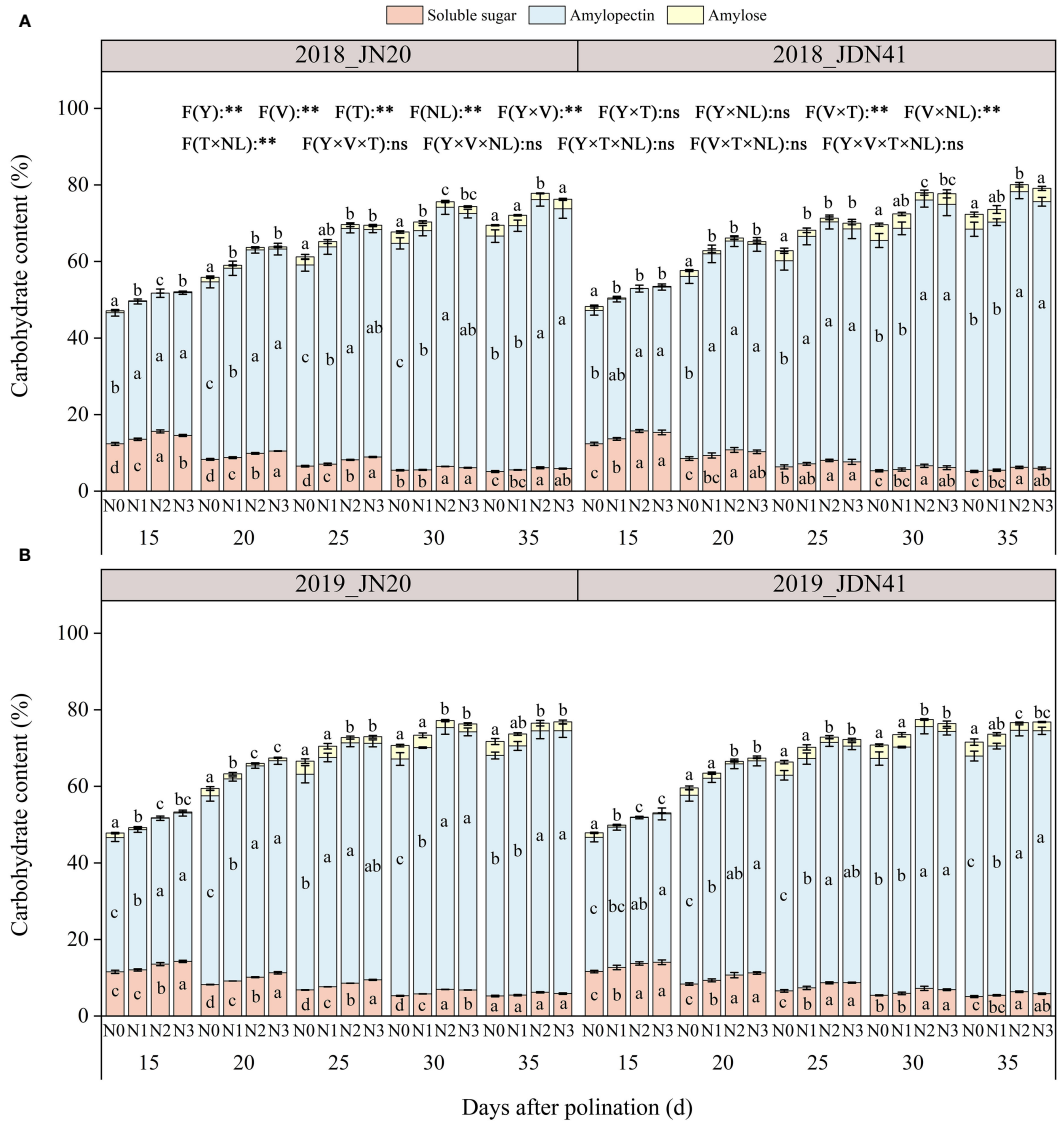
Effects of nitrogen rate on carbohydrate content of two waxy maize varieties at different days after pollination in 2018 **(A)**, 2019 **(B)**. ns indicates no significant differences, and * and ** indicate significant differences at *P* < 0.05, and *P* < 0.01 level, respectively. Different lowercase letters indicate significant differences between nitrogen rates at the *P*<0.05 level. The error bar indicates standard error based on 3 data.

To detect the dynamic metabolism of grain C in waxy corn under different N application rates, the contents of three kinds of carbohydrates were determined, including amylose, amylopectin and soluble sugar. The ANOVA analysis showed that the overall content of three carbohydrates was strongly influenced by specific factors and their interactions, as illustrated in **Figure 6**. As shown in **Figure 6**, the total C content of JDN41 was lower than that of JN20 at any time point under four nitrogen levels in two years. As time goes by, the levels of total C, amylose and amylopectin content of both genotypes promoted sharply from 15 to 30 DAP, and almost ceased from 30 to 35 DAP under the four N levels. The soluble sugar content of the two genotypes showed the opposite change trend compared to amylopectin content and amylose content. In addition, the effects of N level on all C components of both genotypes at any grain filling stage were almost consistent within two years, all showing N2≥N3>N1>N0.

In detail, the soluble sugar content of JN20 and JDN41 decreased over two years from 15 DAP to 35 DAP under different nitrogen levels. The amylopectin content in grain increased with time for both JN20 and JDN41 varieties, with JN20 showing an increase from 34.54% to 62.40% at 15 DAP and 35 DAP, respectively, and JDN41 increasing from 35.06% to 62.84% during the same period (**Figure 6**). The amylose content in JN20 grains increased from 0.78%, 0.24%, 0.06%, and 0.19% at 15 DAP to 3.34%, 3.02%, 1.74%, and 2.98% at 35 DAP under N0, N1, N2, and N3. For JDN41, the amylose content increased from 1.19%, 0.54%, 0.10%, and 0.25% at 15 DAP to 3.65%, 3.10%, 2.02%, and 2.30% at 35 DAP. These results demonstrated that increasing N dose had significantly negative effects on the soluble sugar metabolism and positive effects on the amylopectin and amylose biosynthesis in waxy maize grains.

#### 3.3.2 C metabolism enzymes

**Figure 7:**
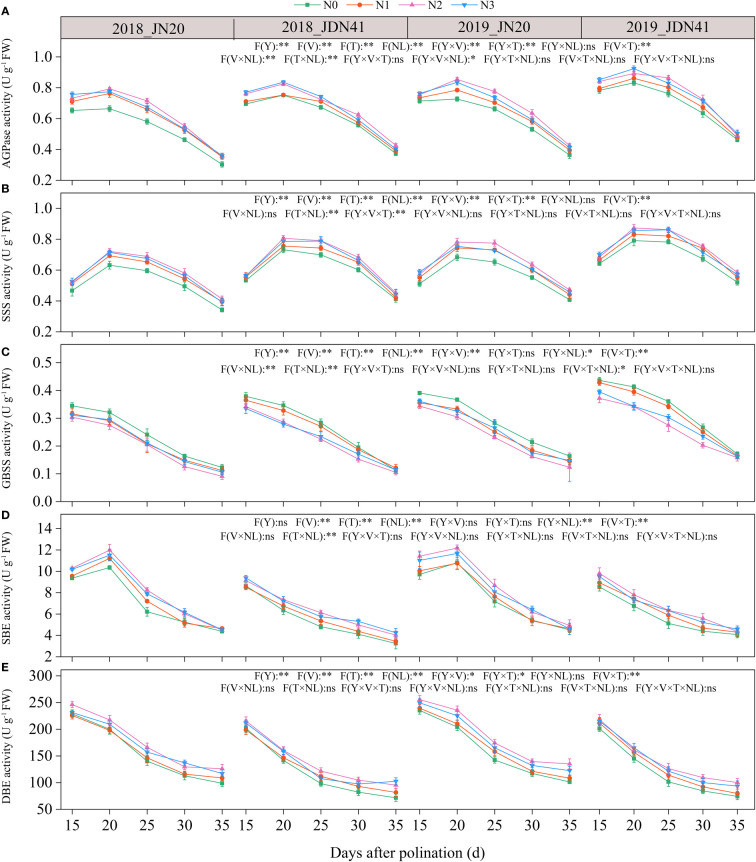
Effects of nitrogen rate on the enzymatic activity of carbon metabolism of two waxy maize varieties at different days after pollination. ns indicates no significant differences, and * and ** indicate significant differences at *P* < 0.05, and *P* < 0.01 level, respectively. The error bar indicates standard error based on 3 data. **(A-E)** ADP-glucose pyrophosphorylase (AGPase) activity, soluble starch synthase (SSS) activity, granule bound starch synthase (GBSS) activity, starch branching enzyme (SBE) activity and starch-debranching enzyme (DBE) activity of two waxy maize varieties at different days after pollination in 2018 and 2019, respectively.

Five C metabolism enzymes in the waxy corn grains, including soluble starch synthase (SSS), granule bound starch synthase (GBSS), starch branching enzyme (SBE), starch debranching enzyme (SDBE) and ADP-glucose pyrophosphorylase (AGPase) were determined. Significant variations in the activities of SBE and DBE enzymes were seen across genotypes throughout different time points, nitrogen application rates, and years. However, the activities of AGPase and SSS only displayed noticeable differences between the two genotypes in 2019 (**Figure 7**; **Supplementary Figure 7**). Over time, the activities of GBSS and DBE declined progressively, while the activities of AGPase, SSS, and SBE increased from 15 to 20 DAP and then fell gradually. Furthermore, the application of N enhanced the activity of AGP and SSS, while it suppressed the activity of GBSS and SBE. The impacts of nitrogen application on DBE activity were relatively complex. The N effects for DBE activity of both genotypes were not significant from 15 to 25 DAP in 2018, but applying N apparently enhanced the activity of DBE of both genotypes from 25 to 35 DAP. Meanwhile, N application had a significant impact on DBE activity of both genotypes in 2019. For example, the JN20’s AGPase activity raised by 1.91%-20.85%, 4.36%-26.82%, and 1.29%-29.68% with N1, N2, and N3 treatments compared to N0 over the five grain filling stages over two years, respectively. The JDN41 values increased by 0.15%-14.53%, 0.14%-17.53%, and 1.51%-17.66%, respectively. In contrast, the GBSS activity of JN20 under N1, N2 and N3 treatments diminished with ranges of 0.64%-23.01%, 7.21%-58.81% and 2.57%-24.34% compared to N0 over the five grain filling stages within the two years, respectively. And that of JDN41 dropped with ranges of 0.26%-14.59%, 5.56%-34.26% and 2.42%-28.70%, respectively.

#### 3.3.3 Regression analysis

Regression analysis results showed that both JN20 and JDN41 GNC were significantly positively correlated with the soluble sugar content, and R^2^ of which was 0.80 and 0.79, respectively (**Supplementary Figure 8**). However, both JN20 and JDN41 GNC were significantly negatively correlated with the amylose content (R^2^ of 0.65 and 0.73, respectively) and amylopectin content (R^2^ of JN20 and JDN41 was 0.47 and 0.42, respectively).

**Figure 8:**
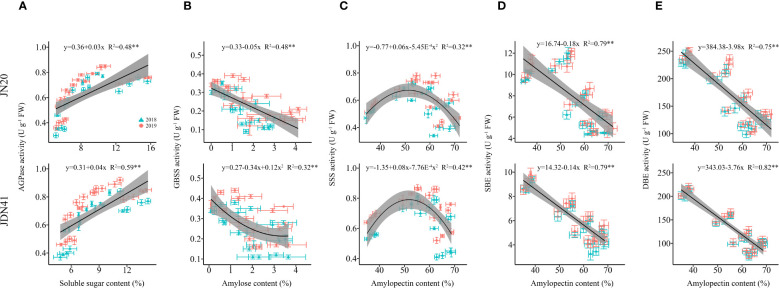
Relationship between soluble sugar content and ADP-glucose pyrophosphorylase (AGPase) activity **(A)**, amylose content and granule bound starch synthase (GBSS) activity **(B)**, amylopectin content and soluble starch synthase (SSS) activity **(C)**, amylopectin content and starch branching enzyme (SBE) activity **(D)**, and amylopectin content and starch-debranching enzyme (DBE) activity **(E)** of two waxy maize varieties at different days after pollination. ns indicates no significant differences, and * and ** indicate significant differences at *P* < 0.05, and *P* < 0.01 level, respectively.

Considering that SSS, SBE and SDBE participate in amylopectin synthesis, GBSS plays crucial role in amylose synthesis and AGPaseis is responsible for converting soluble sugar to starch (Smith and Zeeman, 2020). Therefore, we further analyzed the linear relationship between the relevant enzymes and carbohydrates (**Figure 8**). It showed that AGPase activity and soluble sugar content had a highly significant positive correlation in both genotypes, and SBE and DBE activity of both genotypes had extremely significant negative correlation with amylopectin content, while GBSS activity and amylopectin content was significantly correlated only in JN20. Furthermore, the correlation between SSS activity and amylopectin content was a univariate quadratic relationship, showing a positive correlation between SSS activity at low amylopectin content and a negative correlation at high amylopectin content.

### 3.4 Effects of nitrogen level on the dynamic C/N ratio

**Figure 9:**
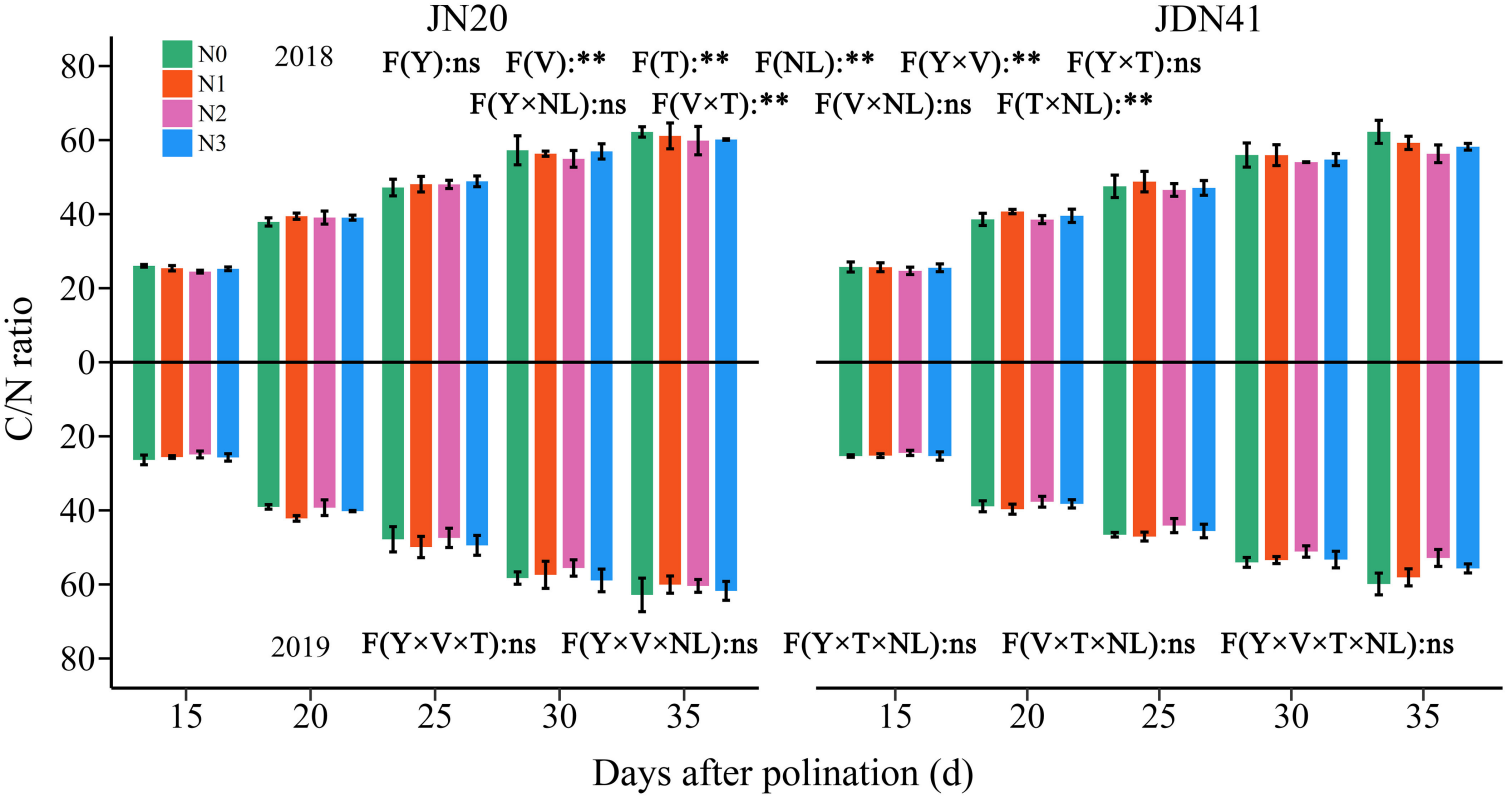
Effects of nitrogen rate on grain C/N ratio of two waxy maize varieties at different days after pollination. ns indicates no significant differences, and * and ** indicate significant differences at *P* < 0.05, and *P* < 0.01 level, respectively. The error bar indicates standard error based on 3 data.

The ANOVA analysis revealed that C/N ratio in waxy corn grains was significantly influenced by V, T, NL, Y×V, V×T and T×NL, but Y and other interactions between the two or three factors had no significant effects on the C/N ratio (**Figure 9**). The C/N ratio of both genotypes was similar at same time point and under same N levels within two years. With time, the C/N ratio of both genotypes increased gradually under the four N levels within two years, indicating that the N accumulation in waxy corn grains occurs earlier than the C accumulation. In comparison, the C/N ratio of JN20 and JDN41 at 20, 25, 30, and 35 DAP gradually grew by an average of 32.49% and 35.78%, 54.16% and 55.13%, 75.47% and 74.26%, and 87.09% and 84.25% compared to 15 DAP, respectively, over the four N treatments within two years.

In addition, it showed that applying N had no effect on the C/N ratio at the early grain filling stages (15-25 DAP), but it caused a decrease in the C/N ratio at the later grain filling stages (30-35 DAP) (**Figure 9**). In detail, the JN20 C/N ratio under N1 and N3 treatments increased with ranges of 0.27%-9.56%, and 0.40%-12.35% compared to N0 over the five grain filling stages within the two years, respectively. And the C/N ratio of JDN41 under N1 and N3 treatments increased with ranges of 0.31%-11.28%, and 0.12%-7.95% compared to N0 over the five grain filling stages within the two years, respectively. However, the C/N ratio of JN20 and JDN41 under N2 treatments decreased with ranges of 0.17%-7.79%, and 0.09%-14.50% compared to N0 over the five grain filling stages within the two years, respectively.

### 3.5 Structural equation model analysis

**Figure 10:**
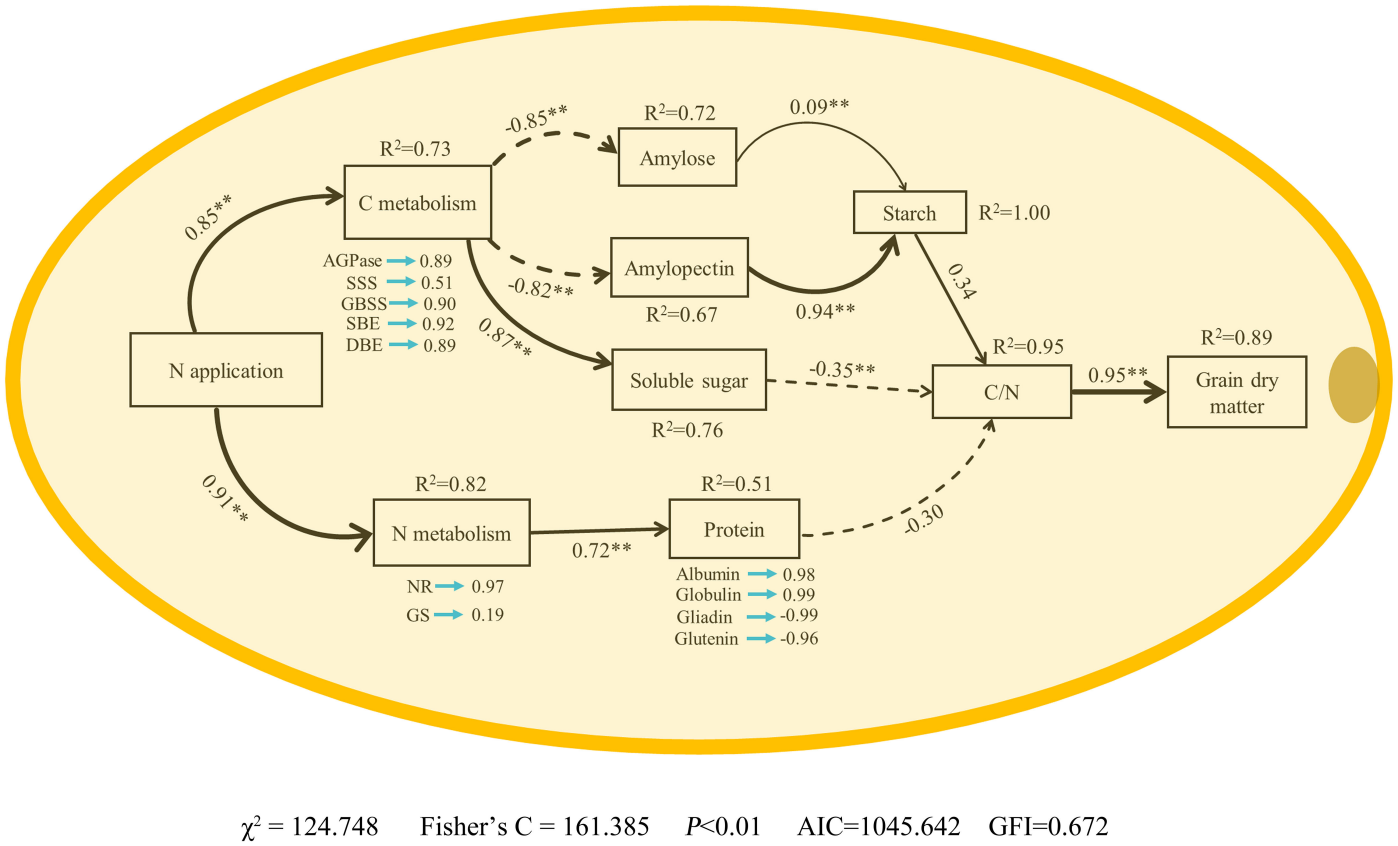
Structural equation model analysis (SEM) shows N fertilizer supply increased waxy maize grain dry matter–Effects of nitrogen fertilizer on enzyme activities of carbon and nitrogen metabolism, starch, soluble sugar and protein contents, C/N, dry matter in waxy maize grains. NR, nitrate reductase; GS, glutamine synthetase; AGPase, ADP-glucose pyrophosphorylase; SSS, soluble starch synthase; GBSS, granules bound starch synthase; SBE, starch branching enzyme; DBE, starch-debranching enzyme. The R^2^ values denote the proportions of variance explained by relationships with other variables in SEM. Single-headed arrows indicate the hypothesized direction of causation. Indicated values denote the standardized path coefficients of a positive (positive values) or negative (negative values) effect. Solid and dashed lines represent positive and negative paths, respectively. The width of the black arrows indicates the strength of the causal relationship. * and ** represent *P* < 0.05 and *P* < 0.01, respectively.

SEM analysis showed that N application rate had significant positive influences on the enzyme activities related to C and N metabolism at 15-35 DAP directly (**Figure 10**). The enzymatic activities associated with carbohydrate metabolism exerted a markedly negative impact on the contents of amylose and amylopectin, whilst manifesting a significantly positive influence on the concentration of soluble sugars. In contrast, the contents of amylose and amylopectin were found to significantly bolster the overall starch content. Furthermore, the enzymatic processes integral to nitrogen metabolism were observed to substantially enhance protein levels within the grain. Interestingly, the starch content demonstrated a pronounced positive correlation with the carbon/nitrogen (C/N) ratio within the grain, whereas the concentrations of soluble sugars and proteins exhibited a notably adverse effect on this ratio. The C/N ratio, in turn, was significantly allied with an increase in grain dry weight. Therefore, we posit that the application of nitrogen serves as a catalyst in elevating grain dry weight through its nuanced modulation of the metabolic pathways governing carbon and nitrogen in waxy corn grains, underpinning a multifaceted influence on the intricate dynamics of grain composition and weight augmentation.

## 4 Discussion

### 4.1 The appropriate nitrogen application rate promotes nitrogen metabolism in waxy maize kernels

Nitrogen (N) is a fundamental nutrient that plays pivotal roles in the synthesis of proteins, amino acids, and other vital compounds in plants (Yue et al., 2021). The appropriate N application is crucial for optimizing maize yield, kernel quality and nutritional value (Wei et al., 2017). The quantity of N present in kernels is mainly conditioned by amounts of N remobilization from reserves accumulated in the leaves and stems (Dordas, 2009; Masclaux-Daubresse et al., 2010). A recent study reported that maize kernel N accumulation dynamics were highly positive regulated under 84, 168 and 224 kg N ha^−1^ compared to none N application, and the effects increased with the increasing rate of N application (Olmedo Pico and Vyn, 2021). Similarly, a study in rice and maize indicated that the grain N content increased with N increment, which were conducted under 0 to 240 kg N ha^−1^ with a 40 kg N ha^−1^ frequency (Mondal et al., 2023). In contrast, several studies have demonstrated that the effects of N application on grain quality are highly dose-dependent, reasonable fertilization is one of the most effective measures to improve maize quality, but excessive N deteriorated the grain quality (Rossini et al., 2011; Zhou et al., 2020; Wu et al., 2023). In present study, we found that N dose had significantly positive effects on kernel N content in two waxy maize genotypes (JN20 and JDN41), and N2 (240 kg ha^-1^ N) achieved the highest kernel N content with an increase range of 5.92-19.48% compared to N0 over 15-35 DAP within two years, indicating that reasonable N application was necessary to stabilize kernel N content in waxy corn (**Figure 2**). At the same time, the kernel N content in JN20 and JDN41 decreased constantly with averages of 1.49% and 1.56% to 1.21% and 1.29% from 15 DAP to 35 DAP, respectively, based on four N treatments within two years (**Figure 2**), it means that translocation and accumulation of N in grains should take priority over the carbohydrate (C) accumulation.

The grain protein content (GPC), comprised of gliadins and glutenins (storage proteins), as well as albumins and globulins (metabolic proteins), is an important determinant of grain quality in maize. In wheat, increasing nitrogen application resulted in higher concentrations of total protein in the grains (Monostori et al., 2017), which largely relying on the accumulation of gliadins and glutenins (Fuertes-Mendizábal et al., 2013). However, another study demonstrated that a N rate of 240 kg ha^-1^ was sufficient to satisfy N uptake requirements from soil, and maintain protein accumulation in wheat grains (Zhang et al., 2017). In this study, we detected that the albumin content and globulin content in the grains of two waxy maize varieties under four N levels all decreased gradually from 15 to 35 DAP, while that of gliadin content and glutelin content showed a continuously increasing trend, and the gliadin and glutelin content of both genotypes almost reached the highest values under N2 treatment, especially at the later grain filling stage (**Figure 5**).

Nitrate reductase (NR) and glutamine synthetase (GS) are the major enzymes participating in the process of N assimilation (Liu et al., 2022). NR is the first enzyme in the system of transforming inorganic nitrogen into organic nitrogen that would limit the overall nitrogen assimilation in plants (Hageman et al., 1962), and re-assimilation of ammonium derived from protein degradation is determined by GS (Bernard et al., 2008). In this study, the impacts of N dose on NR and GS activities were similar as that on kernel N content, and an appropriate N application rate play important roles in resisting the decrease of the activities of NR and GS during grain filling stage to ensure the N metabolism (**Figures 3**, **4**). The regression analysis indicated that grain N content was significantly positively correlated with the NR and GS activity, total protein content, gliadin content and glutelin content, but was negatively correlated with albumin content and globulin content (**Supplementary Figures 5**, **6**). Thus, we propose that appropriate applying N can promote activities of nitrogen metabolism enzymes to induce gliadin and glutelin biosynthesis the in waxy maize kernels.

### 4.2 The reasonable application of N induces C biosynthesis

N is also the main component of chlorophyll, which affects the accumulation of leaf biomass and the efficiency of photosynthesis (Wani et al., 2021), and almost C is synthesized in plants through photosynthesis. Therefore, N is crucial for carbohydrate metabolism. Hence, N is crucial for carbohydrate metabolism. Numerous studies have investigated the effects of N fertilization on carbohydrate accumulation in maize kernels and have shown that increasing N application during the grain filling phase stimulates the accumulation of starch and soluble sugars in maize kernels. Nitrogen can promote the transport of C to grains (Vijayalakshmi et al., 2013; Ma et al., 2023). It was reported that N fertilizer could promote starch accumulation in wheat, and higher amylopectin and total starch contents were obtained when N level was 240 kg ha^-1^ (Li et al., 2013). However, another study showed that the amylose, amylopectin and total starch contents in wheat gradually decreased with increasing N content, while the starch accumulation rate increased significantly (Lv et al., 2021). As *waxy* gene mutation (*Wx*), waxy corn kernels contain approximately 95–100% amylopectin (Zhou et al., 2016). In this study, amylose was also detected in the grains of two selected waxy maize varieties, and its content ranged from 0.03% to 4.13% over four N levels and five grain filling stages within two years (**Figure 6**). At the same time, the effects of N level on soluble sugar, amylose and amylopectin contents of both genotypes at any grain filling stage within two years were almost similar, all showing N2≥N3>N1>N0 (**Figure 6**). The soluble sugar content in the grains of JN20 and JDN41 under N2 increased by an average of 23.74% and 26.42%, compared to N0, respectively, over the five grain filling stages within the two years, and that of the amylopectin content increased by an average of 13.00% and 10.30%, respectively, but that of the amylose content decreased by an average of 59.26% and 61.39%.

In general, ADP-glucose pyrophosphorylase (AGPase), granule bound starch synthase (GBSS), starch debranching enzyme (DBE), soluble starch synthase (SSS) and starch branching enzyme (SBE) are considered to be the five key enzymes involved in starch synthesis and metabolism, regulating the process of starch synthesis and accumulation (Yi et al., 2014). Studies have shown that N availability directly influences the activity of enzymes involved in C metabolism, thereby affecting C accumulation in plant tissues (Krapp et al., 2011). In rice, the application of nitrogen fertilizer at the appropriate rate enhanced C biosynthesis by promoting the activity of key enzymes in the glycolysis and starch synthesis pathways (Xu et al., 2012). N fertilization can affect grain growth mainly by regulating starch synthesis in endosperm, and one of these effects is achieved through altering enzyme activity (Singletary et al., 1990). There have been many studies about the key enzyme activity in wheat, rice and potato as affected by applying N fertilizer. In wheat, N application significantly increased the activities of the GBSS and SSS in wheat, and the starch accumulation rate was higher under higher N level (Wang et al., 2014). In rice, the activities of AGPase, SSS, GBSS and SBE were significantly increased after given N supply (Li et al., 2008). In contrast, the activities of the AGPase and SSS in potato significantly increased under low N level and then decreased with increasing N level, while the activities of the GBSS and SBE did not respond significantly to N fertilization (Whiley et al., 1989). In this study, we detected dynamic changes of the activities of AGPase, SSS, GBSS, SBE and SDBE in grains of two waxy maize varieties after applying different N rates. We found that applying N could enhance the AGP and SSS activity, but had an inhibitory effect on the activities of GBSS and SBE. The correlation analysis results indicated that grain N content was significantly positively correlated with the soluble sugar content, but negatively correlated with the amylose content and amylopectin content (**Supplementary Figure 7**). In addition, AGPase activity and soluble sugar had a highly positive correlation, and SBE and DBE activity had significant negative correlation with amylopectin content (**Figure 8**). Above all, we propose that appropriate applying N can regulate the activities of related C metabolism enzymes to induce C biosynthesis the in waxy maize kernels.

### 4.3 Coordination of N and C improves dry matter accumulation of waxy maize kernels

C metabolism provides the necessary energy and C skeletons for reduction of NO_3_^-^ and synthesis of amino acids and, therefore, improving C metabolism increases N metabolism enzyme activities, assimilation of NH_4_^+^, and efficiency of N metabolism in crops (Zhang et al., 2023). Improving the intensity and coordination of C and N metabolism in maize is particularly important for improving yield. This study showed that applying N had no effect on the C/N ratio in waxy maize grains at the early grain filling stages, but it caused a decrease in the C/N ratio at the later grain filling stages. Structural equation model (SEM) analysis showed that N application rate could positively affect the enzyme activities related to C and N metabolism to regulate the carbohydrate and protein content in waxy maize grains, resulting in coordinating the C/N ratio, to determine the grain dry matter accumulation (**Figure 10**). Similarly, a recent study found that optimal N application rate and nitrate-to-ammonium N ratio treatment enhanced key enzyme activities of C and N metabolic pathways, led to increase nonstructural carbohydrate accumulation, insufficient N supply reduced grain weight, and excessive N supply caused a reduction in the C/N ratio, reducing the export of photosynthesis products and negatively affecting seed formation. These results indicated that applying N promoted the grain dry weight by affecting the processes of waxy corn grain C and N metabolism.

### 4.4 Reasonable nitrogen application rate ensures the formation of maize yield

As the waxy corn is mainly consumed as fresh food, so the effects of N application rate on its quality and yield seem like the hot research projects. Applying N fertilizer at an optimal rate is essential for ensuring the formation of maize yield. Insufficient N application negatively impacts N reactivation and recycling, injures the leaves, reduces in chlorophyll content and photosynthesis activity (Bassi et al., 2018; Mu et al., 2018; Mu and Chen, 2021), ultimately significantly affects grain formation and weight (Nasielski et al., 2019; Santos et al., 2023). Conversely, excessive N results in early-stage overgrowth, increases the risk of lodging, and reduces NUE, ultimately affects yield formation (Hammad et al., 2022; Shao et al., 2024). In present study, we found that N dose had significantly positive effects on kernel dry matter accumulation in two waxy maize genotypes, and N2 achieved the highest grain dry weight (GDW) with an increase range of 9.88-34.20% compared to N0 over 15-35 DAP within two years (**Figure 1**), indicating that reasonable N application was necessary to stabilize GDW in waxy corn. As discussed above, the carbon and nitrogen metabolism in waxy maize kernels were enhanced under N2 treatment compared to low N level (N0 and N1) and high N level (N3), leading to the highest accumulation of kernel dry matter. Therefore, for waxy corn, N2 may be a reasonable N application amount.

## 5 Conclusions

In this study, we found that appropriate application of N promoted the activity of NR and GS to regulate kernel N remobilization, and induced the activities of the enzyme activities related to C metabolism (AGPase, SSS, GBSS, SBE and DBE) to enhance C and N accumulation in the grains of JN20 and JDN41, and to obtain more dry matter at accurate harvest period under N2 treatment. It is worth noting that grain dry matter was significantly positively correlated with C/N ratio, suggesting that the increase of starch content induced grain dry matter. Therefore, reasonable applying N could coordinate the metabolism of C and N in grain during the grain filling stages, which was important to regulate the formation of grain dry weight. Our results highlight the great potential of N fertilizer dose in influencing the C and N accumulation in the grains of waxy maize, which provides a reference for the appropriate application of nitrogen fertilizer and the selection of cultivation management practices for waxy corn production.

## Data availability statement

The original contributions presented in the study are included in the article/**Supplementary material**. Further inquiries can be directed to the corresponding authors.

## Author contributions

WF: Data curation, Formal analysis, Funding acquisition, Investigation, Methodology, Project administration, Visualization, Writing – original draft, Writing – review & editing. WX: Formal analysis, Software, Visualization, Writing – original draft. ZZ: Data curation, Formal analysis, Resources, Visualization, Writing – review & editing. ZS: Conceptualization, Investigation, Writing – review & editing. WW: Conceptualization, Investigation, Writing – review & editing. YB: Writing – original draft. HW: Writing – original draft. PQ: Investigation, Writing – review & editing. JX: Funding acquisition, Supervision, Validation, Writing – review & editing. BC: Conceptualization, Supervision, Validation, Writing – original draft.
