# Supplementary figures and images for: Nitrogen fertilizer application rate affects the dynamic metabolism of nitrogen and carbohydrates in kernels of waxy maize
Source: Front Plant Sci. 2024 Aug 1;15:1416397. doi: 10.3389/fpls.2024.1416397 (PMC11324447; doi:10.3389/fpls.2024.1416397)

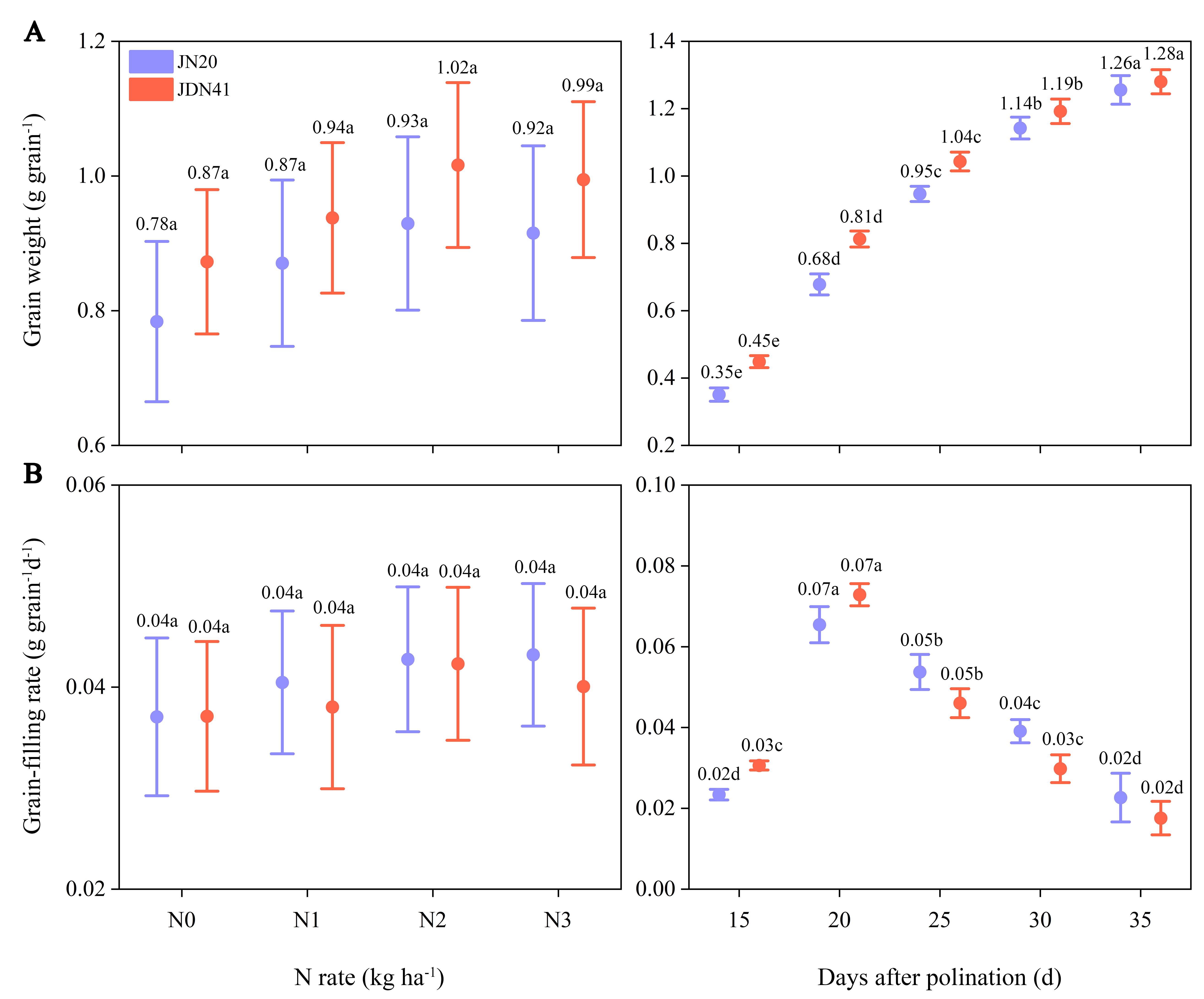

Supplement: Supplementary file 1 [file Image_1.tif]

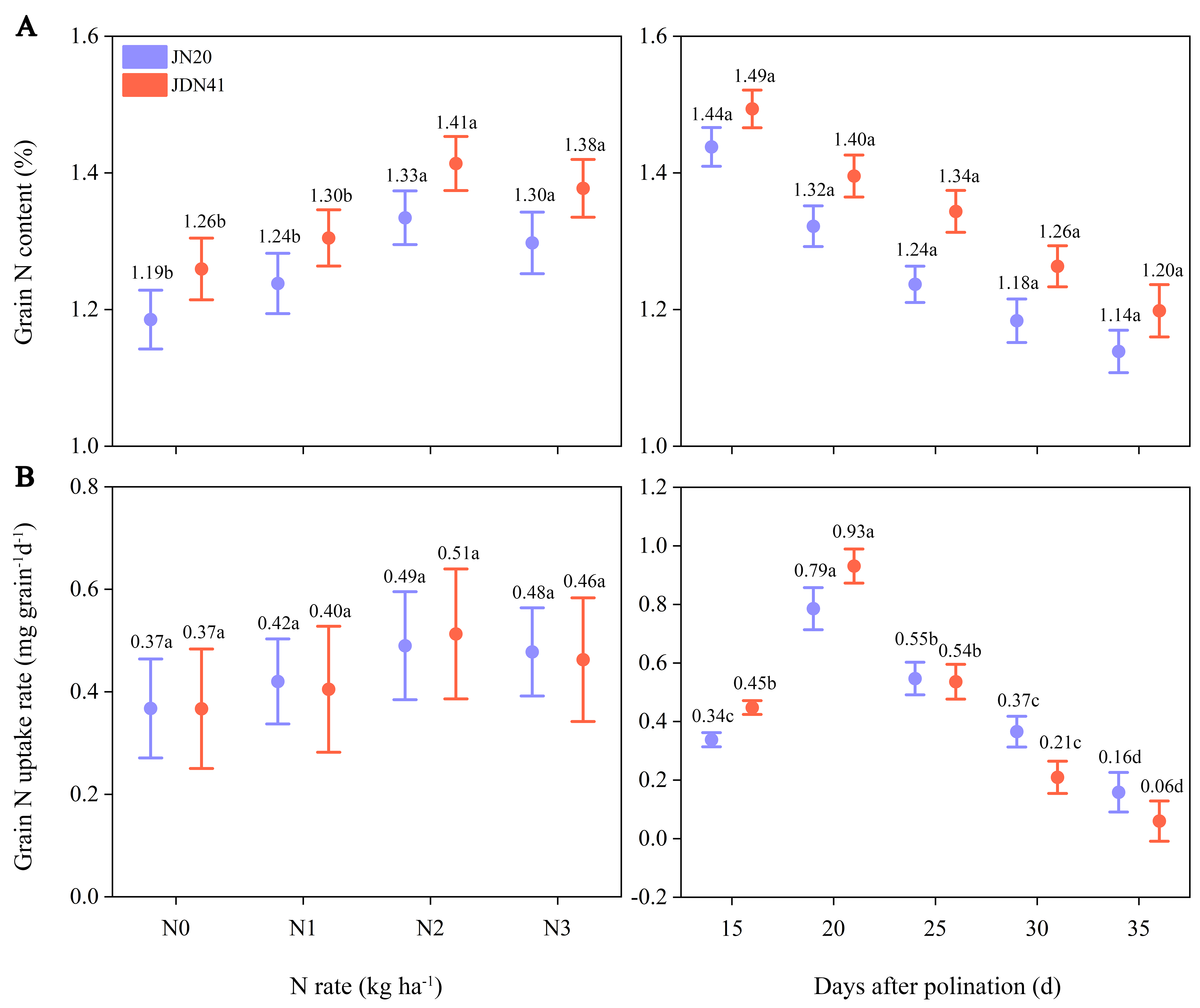

Supplement: Supplementary file 2 [file Image_2.tif]

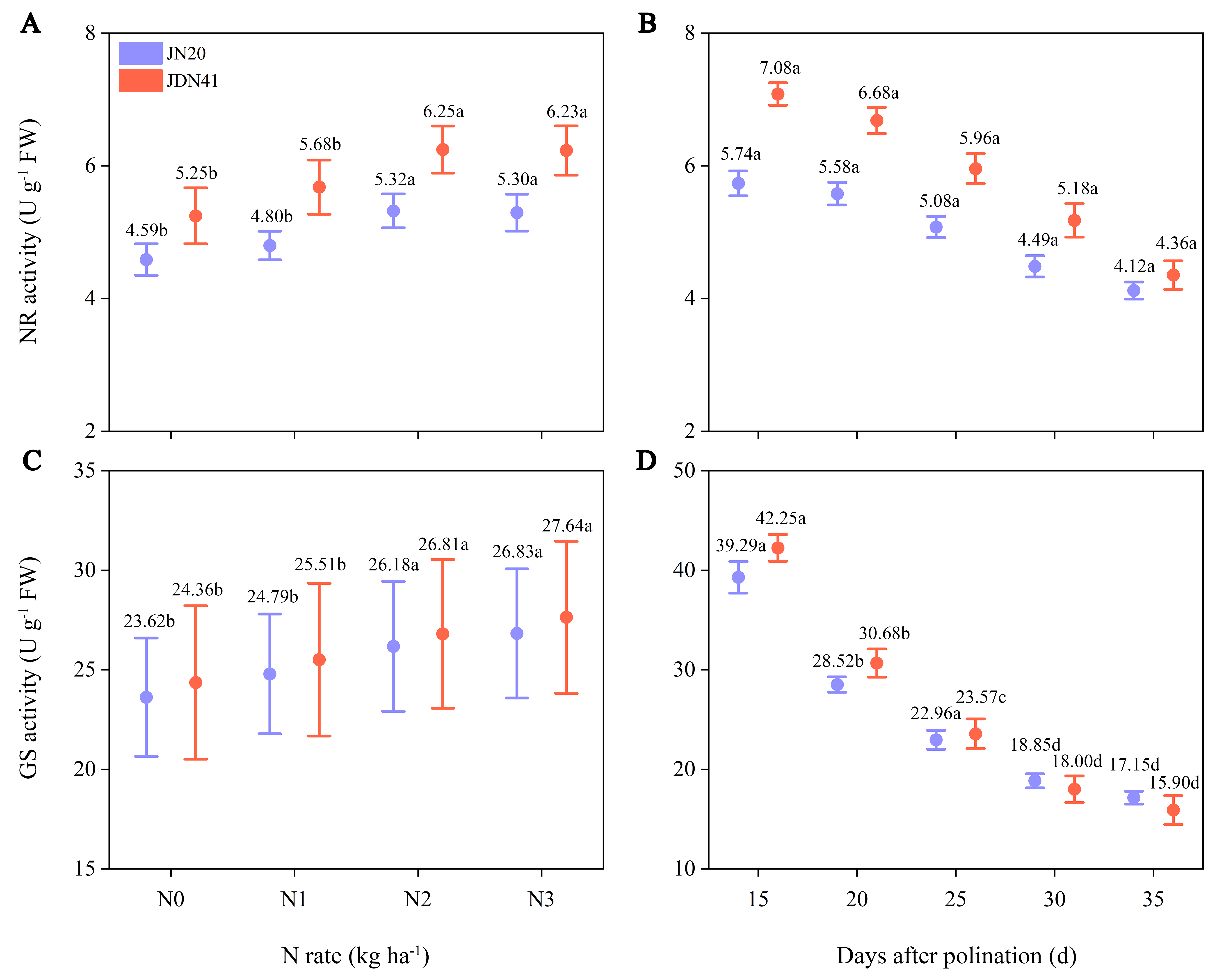

Supplement: Supplementary file 3 [file Image_3.tif]

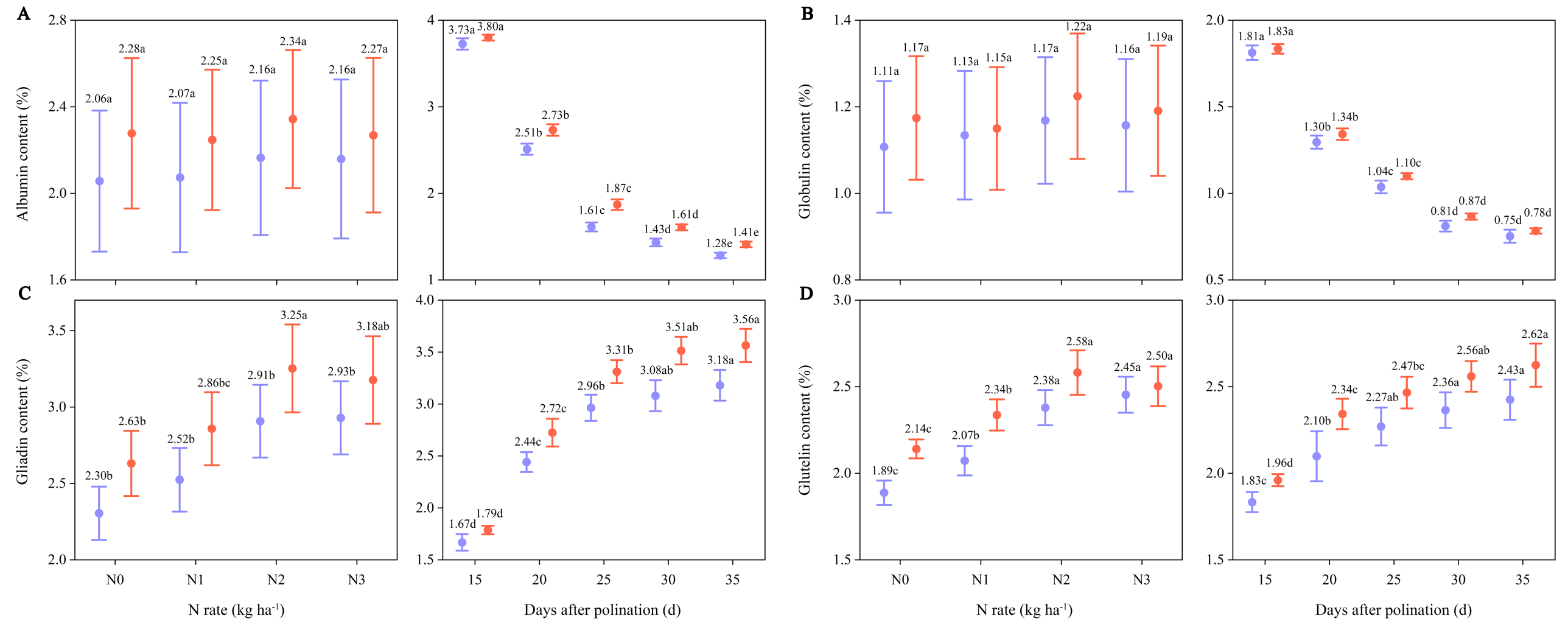

Supplement: Supplementary file 4 [file Image_4.tif]

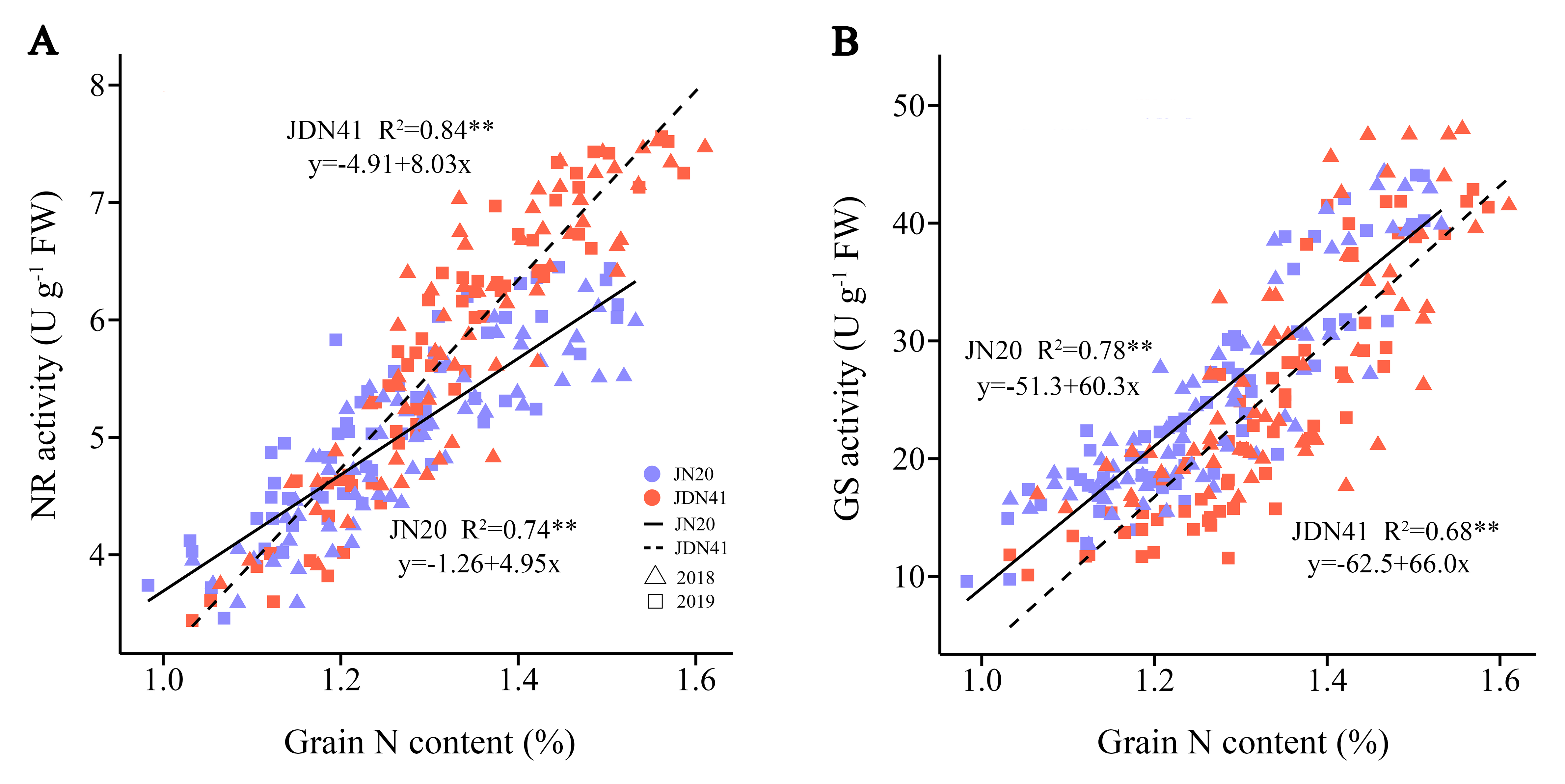

Supplement: Supplementary file 5 [file Image_5.tif]

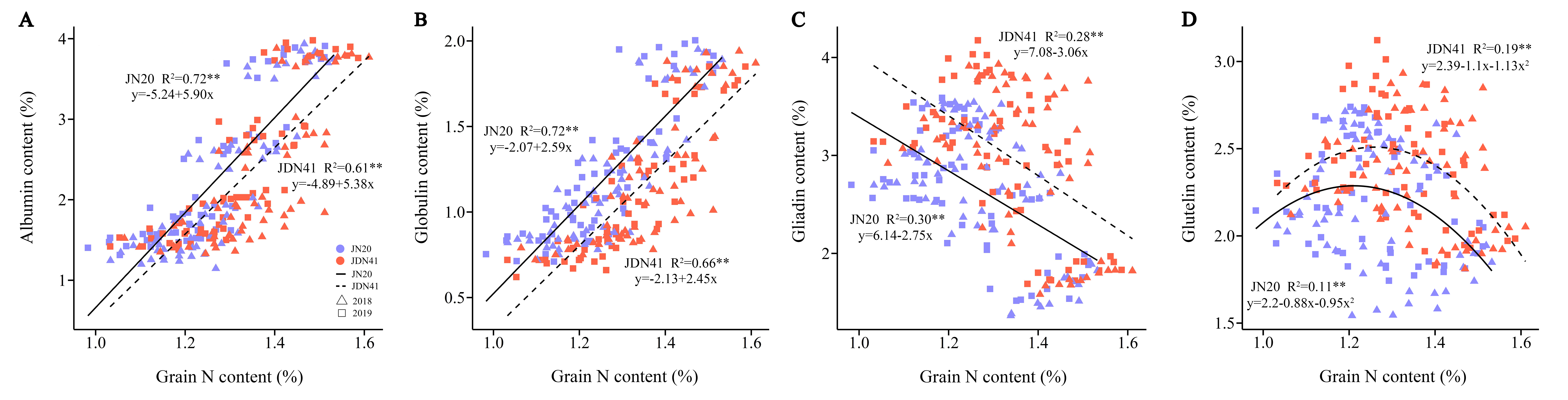

Supplement: Supplementary file 6 [file Image_6.tif]

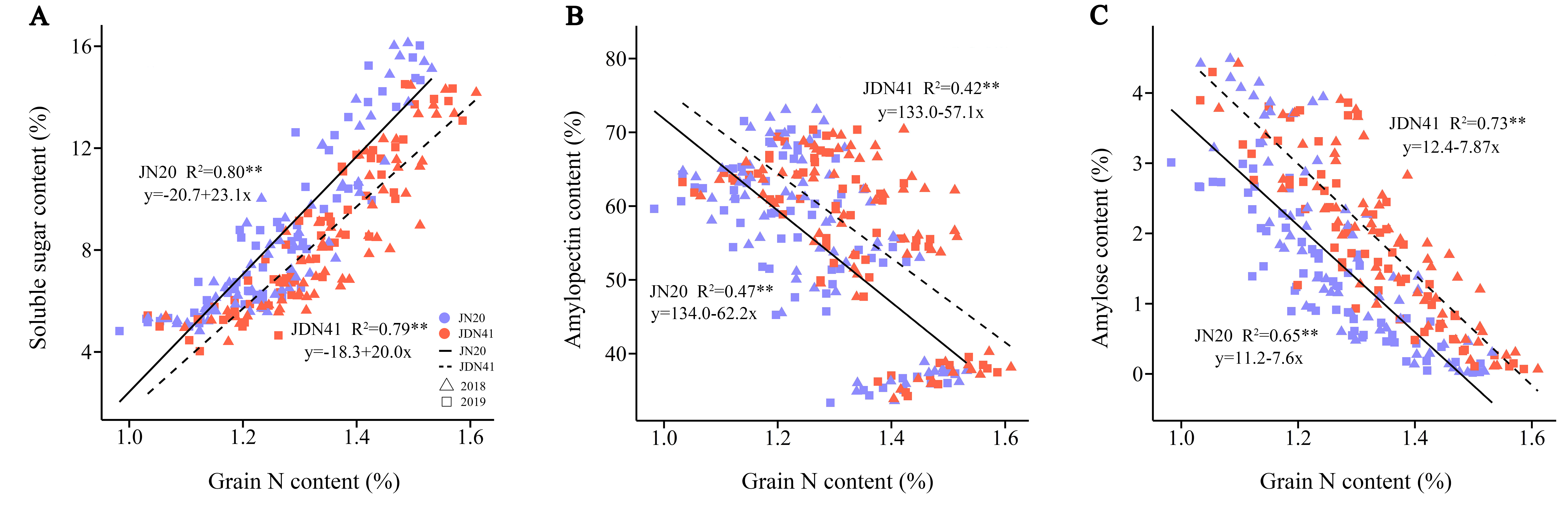

Supplement: Supplementary file 8 [file Image_8.tif]
